# Gradually Guiding Nursing Students through Their Capstone Course: Registered Nurse Preceptors Share Their Experiences

**DOI:** 10.1155/2011/645125

**Published:** 2011-04-14

**Authors:** David L. Martin, M. Kathleen Brewer, Nancy Barr

**Affiliations:** The University of Kansas School of Nursing, 3901 Rainbow Boulevard, Mail Stop 4043, Kansas City, KS 66160-7502, USA

## Abstract

Professional precepted immersion courses (capstone) have become the standard as a means to prepare senior nursing students to enter the workforce. Preceptors have a significant role in developing the student nurse, yet exactly how to prepare preceptors for this role has been an ongoing discussion. This qualitative inquiry explored the educational needs of clinical registered nurse (RN) preceptors who work directly with senior nursing students in a professional precepted immersion (capstone) course. A descriptive qualitative design was used to examine preceptors responses to a prepared set of questions about their educational needs. Results showed that preceptors have three distinct sets of learning needs: the need to know the expectations of their role, wanting to know how best to role model for the student, and knowing how to socialize the student into the profession of nursing. Overall, preceptors communicated their desire and commitment to doing the best job possible. They also clearly stated their expectation of faculty to have a physical presence on the nursing unit that included being proactive in resolving mismatches and exposing the student to the roles of provider of care, leader and manager of care, and member of profession.

## 1. Introduction

Professional precepted immersion courses (Capstone) have become the standard as a mechanism to prepare senior nursing students to enter the workforce. The primary guide in this process is the students' preceptor. Preceptors are registered nurses (RNs) who often have volunteered for the role and are viewed as experts in their clinical area. Preceptors have a significant role in developing the student nurse. How to prepare preceptors for this role is a subject of ongoing debate within the profession of nursing warranting investigation.

## 2. Course Description

The capstone course in this study was implemented in the Spring semester, 1998. It has a longstanding successful history of pairing senior nursing students with RN preceptors. The course is required of all students immediately prior to graduation. Preceptors are RNs in clinical staff settings in various agencies in the community. These agencies include hospitals, long-term care, and community settings. Senior students select their site and preceptor from offerings developed by the faculty in collaboration with available agencies. Students spend 320 hours in this clinical course, with approximately 16–24 hours of this total spent in clinical seminars and other approved activities. The remainder of the time is spent in the clinical area with the preceptor, where the student transitions into the role of professional nurse. All clinical sites are areas of potential employment after graduation and licensure.

## 3. Review of Literature

### 3.1. Models of Preceptor Course Content

The nursing literature informs the profession about the unique learning needs of RN preceptors working with a newly graduated registered nurse. Much of the literature presents information about the learning needs of preceptor's interactions with employees. Minimal information is available about the learning needs of preceptors working with students in their capstone courses.

 Alspach [1] outlined the primary roles of a preceptor for new graduates as being a role model, a socializer, and an educator. This author further subdivided the educator role into four parts: assessor of learning needs, planner of learning experiences, implementer of learning plan, and evaluator of job performance. Similarly, R. M. Meyer and M. C. Meyer [2] reported results of evaluation about an orientation program for new RNs in a hospital setting. Their convenience sample (*n* = 59) of staff RNs revealed the importance of providing a structured educational program for preceptors that prepared them for the role. Hardy and Smith [3] identified the positive use of a structured preceptor education in an ICU setting that matches personality types of the preceptor and the new employee. These researchers used the Myers-Briggs Personality Inventory Profile. Neither Hardy and Smith nor R. M. Meyer and M. C. Meyer provided information about the specific content of their preceptor training course or identified the needs of the new employee. 

 Santucci [4] discussed the importance of having a preceptor development course for nurses in the acute care setting. The author identified clinical and interpersonal skills, reshaping of values, employer expectations, and preceptor development as important outcomes for a new graduate completing an orientation. Santucci identified preceptor development, assimilating role responsibilities, principles of adult learning, communication skills, teaching techniques, assessment of competency, providing feedback, and facilitating networking all as important components necessary in an orientation program. Neumann et al. [5] outline the content for a three tiered preceptor development course. This model provides content for the individual preceptor which is based on their developed competencies. New preceptors (first level) receive content: in learning styles, needs assessment, feedback, and role modeling. The second level focuses on reality shock, principles of adult education, clinical teaching, and issues related to being a preceptor. The third level stresses role enhancement, networking, and problem solving for the preceptor. Charleston and Goodwin [6] provided insight into the learning needs of preceptors working with professional nurses in a mental health setting. Content of this preceptor course included models of supervision, theoretical perspectives of teaching and learning, dynamics of the preceptor-new employee relationship, curriculum issues in mental health, competency-based education, practice standards, learning outcomes and assessment, process of organizational change, and leadership. The importance of this topic is highlighted by Zilembo and Monterosso [7] who found that the students perform better when the preceptor is a person they identify as having the positive qualities of compassion, care, and empathy. Central to this process is preceptors that understand their role through ongoing support and preparation. 

### 3.2. Selected Research of Literature Review

Previous researchers have addressed the learning needs of preceptors interacting with new graduates and/or new employees to a nursing unit. Ramritu and Barnard [8] completed a qualitative phenomenological study with six new graduate RNs in Australia who had been out of school and working for less than three months. Their methods included use of a semistructured questionnaire and drawings to collect data related to the idea of competence. They discovered eight general categories: safe practice, limited independence, utilization of resources, time and workload management, ethical practice, performance of clinical skills, knowledgeable, and evolving over time as important learning needs for new graduates.

 Etheridge [9] used a longitudinal qualitative research design with new graduates employed in a medical/surgical acute care setting for nine months after graduation. This method yielded the ideas of developing confidence, learning responsibility, developing relationships with others, thinking critically, and the need for faculty and groups to help them process experiences as vitally important concepts for the new nurse to be able to develop for long-term success.

Zahner et al. [10] described a pilot study that addressed the feasibility of using an online continuing education approach to increase knowledge and self-efficacy in nurse preceptors working with nursing students in acute and primary care settings. Their study contained nine modules that could be completed online at the participant's convenience. They found that this approach was effective in increasing the initial knowledge of the content among the preceptors but that there was a decline in this knowledge among participants over time. Information was provided about course modules content yet no mention was given regarding the source of this content. 

Rogan [11] used a quantitative descriptive design to explore perceptions about preceptor preparation among nurses serving as preceptors for baccalaureate nursing students. Using a modified *Preparation of Nurses Who Precept BSN Students Survey*, the author discovered that the two highest areas of content interest for preceptor preparation were identifying preceptor responsibilities and assessment of students learning needs. Rogan did find that differences exist in preceptors' learning needs based on type of nursing unit and on age/experience of preceptor.

Luhanga et al. [12] conducted a qualitative exploratory, descriptive design to describe preceptors perceived effectiveness of a print-based preceptor manual on their role. This research found common themes: accessible themes, role complexity, partners in precepting, and role development.

Previous researchers have addressed the learning needs of preceptors interacting with new graduates and/or new employees to a nursing unit; however, few were located that specifically explored the experience of preceptors working with nursing students. When viewed collectively, the literature supports the importance of investigating and understanding the learning needs of preceptors of student nurses. Identifying the learning needs of preceptors working with senior nursing students during their practicum (capstone) course warrants further investigation.

## 4. Aim of the Study

The aim of this study was to explore the educational needs of clinical preceptors working with senior nursing students in a capstone course. Historically, capstone courses have been reported as being very positive educational experiences for the students (Rebeschi and Aronson [13]); however, the educational needs of their counterpart preceptors are less known.

## 5. Study Design and Methodology

The study was completed using a qualitative descriptive design. This methodology allows researchers to investigate real-world situations as they occur naturally by exploring and describing individuals' personal perspectives and experiences with a particular phenomenon [14]. It is a systematic and exploratory approach with several specific research characteristics that include (1) the researchers as the primary instruments of data collection, (2) rigorous research methods and data collection that avoid bias resulting from researchers' *a priori *knowledge, (3) ensuring the accuracy of data, and (4) highlighting the perceptions of the participants' experiences with the focus of inquiry. As a means to suspend and bracket the researchers' *a priori *knowledge, qualitative research is atheoretical: no predetermined theoretical framework is used to guide the research question(s) Sandelowski and Barroso [15]. 

With qualitative research, the number of participants recruited for the study cannot be determined ahead of time. Adequacy is attained when sufficient data have been collected and saturation occurs and variation is both accounted for and understood [15–18]. Data are considered saturated when no new information is obtained with additional interviews. For this study, data saturation was reached with twelve participants. 

### 5.1. Participants

With Institutional Review Board (IRB) approval from the researchers' parent institution, participants were *purposefully recruited *from two hospitals in a mid-western city. When conducting qualitative research, participants are recruited based on a particular personal characteristic or experience with the phenomenon under investigation. For this study, registered nurses who had served as preceptors for senior nursing students were recruited and asked to participate in the study. One hospital was a medical center teaching hospital and the second a community hospital. Both hospitals have a Magnet Hospital designation from the American Nurses Association (ANA). All nurse preceptors who had senior nursing practicum (capstone) students from the host school of nursing within the past three years were invited to participate. The inclusion criteria to be eligible to participate in the study were (1) 21 years of age or older, (2) willing to do an audiotaped interview, (3) being able to write and speak English, (4) willing to complete demographic data questionnaire, (5) willing to sign informed consent, and (6) serving as a preceptor for a senior nursing student during the student's capstone course. Each preceptor interviewed for the study was given a $25.00 gift card to thank them for their participation. Knowledge of the gift card was known to the preceptor prior to their initial consent.

All participants completed a demographic data form and participated in a semistructured audiotaped interview with one of the investigators (see the appendix in the Supplementary Material available online at doi: 10.1155/2011/645125). True to the methodology of descriptive qualitative research, interviews were conducted at a time and place chosen by the participant. Participants are more apt to feel comfortable sharing their experience if they are allowed to select a setting in which they feel most comfortable. The participants were asked to share their individual experiences with senior nursing students during their capstone course. The investigators asked the participants to describe their perceived learning needs as a preceptor working with senior nursing students during their capstone course. In addition to the central research question, questions designed to probe beyond a superficial level were developed to elicit and illuminate the learning needs of the preceptors. These questions focused on the three major roles of a preceptor, namely, role model, professional socialize, and educator [1]. Participants were recruited and interviewed until data saturation was achieved. All interviews were transcribed verbatim and reviewed first independently and then collectively by the investigators.

## 6. Data Analysis

Qualitative data analysis calls for the investigators to become immersed in the data [19]. The data analysis method developed by Diekelmamnn and Allen [20] was used to analyze the transcripts. The text of the first complete interview was read and re-read by the investigators to get a sense of the experience for the individual. Next, the researchers performed a thematic analysis to uncover the themes hidden in the description of the experiences as stated by the individuals. The investigators probed through the data and searched for common themes, or essences, as a way to obtain insight into the phenomenon. During the analysis, vernacular phrases, thematic statements, and descriptive words were identified that characterized the phenomenon under investigation. These procedural steps were used to analyze each participant's description of their experience. Using the constant comparative method for data analysis [21], the themes and words participants used to describe their learning needs and experience with the student were compared and contrasted with the descriptions given by other participants. This allowed the researchers to make connections between the experiences of individual participants and to get a sense of the whole experience. A final text, using thick descriptions of the experience, was written and included excerpts from the individual interviews. 

### 6.1. Data Analysis and Methodological Rigor

In descriptive qualitative research, scientific rigor is assessed in terms of trustworthiness. Conducting human science research, the researchers-as-instrument are the guardians and defenders of the truth value of the study. The truth value of the research is judged by the exactness and rigor illustrated by the research process. The researchers strictly adhered to the criteria for trustworthiness posited by Guba and Lincoln [21], namely, credibility, transferability, dependability, and conformability of the data. The investigators conducted the study in such a manner that the findings of the study may be judged credible and supported by an audit trail. To establish and maintain scientific rigor, an audit trail was established and maintained throughout the research process, including data analysis. This final step in the research process ensures that the research can indeed be deemed scientific and the findings credible.

## 7. Findings and Discussion

### 7.1. Description of the Participants

Twelve registered nurse preceptors participated in the study. All were staff nurses in their respective institutions. The preceptors ranged in age from 28 to 66 years (*M* = 42.3); 75% (9) were female and 25% (3) male. Educational preparation represented was BSN or BA 58% (7), 33% (4), an AND, and one was masters prepared. Their experience in nursing ranged from 4 to 40 years (*M* = 13.4). Experience in their clinical specialty area ranged from 4 to 29 years (*M* = 11.2). Experience in their current position ranged from 2 months to 29 years (*M* = 7.4). Eleven of the twelve (92%) had previously taken a preceptor course, and seven of the twelve (58%) held national certification from their specialty organizations (Table 1).

### 7.2. Thematic Analysis

When viewed collectively, three very distinct themes bubbled to the surface and emerged from the data, namely (1) needing to know, (2) wanting to know, and (3) knowing (see Table 2). The preceptors identified the need to know the expectations of their role, wanting to know how best to role model for the student, and knowing how to socialize the student into the profession of nursing.

**Theme 1 (needing to know)**

This theme identified those expectations the preceptors had of the school of nursing prior to serving as a preceptor. Included in these expectations were participating in goal setting with the student, understanding the learning needs of the students, successful conflict resolution strategies, expected professional behavior on the part of the student in the clinical setting, and information about the school of nursing curriculum. The preceptors needed to know that they would be working with a faculty member who was friendly, knowledgeable, and approachable. Also, it was very important to the preceptor that the faculty member be available to him or her any time the student was in the clinical setting. One preceptor shared:

…*Mostly open communication that I need…cause I do not need to have them (faculty) here all the time, but if I do need them for something I'd like to be able to email them or call them or whatever and it worked. *

The preceptors expected to have sufficient information about the student's past clinical work experience and interest area to be able to design a meaningful clinical experience. They shared that they had a need to be provided with the course objectives and the skill level of the student they were guiding. All preceptors were committed to meeting the expectations of the school of nursing and facilitating a positive and rewarding capstone experience for the student under their tutelage. The preceptors were interested in being engaged in addressing their on-going learning needs of providing appropriate coaching, completing student performance evaluations, and troubleshooting in the clinical setting. One participant thoughtfully offered:

*I expect to know the expectations for the student, kind of broad expectations. And I expect to be given information on how to contact the instructor if there is any reason for that.*

Another preceptor stated:

*I expect the faculty to provide me with the handbook from the facility (School of Nursing) to let me know what their expectations are of the preceptor program. I also would like to know who the student is ahead of time and when they would be starting the practicum with me and what the length of time that would be and what they're scheduled…whether they would be following my schedule or if they have a schedule they may be following.*

**Theme 2 (wanting to know)**

Embedded in this theme was that preceptors wanted to know the best way to be an effective role model for their student. Preceptors acknowledged stumbling somewhat when confronted with the dilemma of allowing the student to be engaged in leading and managing patient care. It is noteworthy to mention that a dichotomy appeared that revealed a distinct difference between the preceptor and the educator. The preceptors did not believe the students needed more information about the roles of the other members of the health care team as they expected the student to have this information prior to beginning their capstone course. Conversely, the faculty expected the preceptor to foster learning experiences between the student and other interprofessional individuals, including physicians, physical therapists, social workers to name but a few.

The preceptors were interested in knowing, from the student, the best way for the student to learn in the clinical environment. The preceptors acknowledged that, while some students learn best from the preceptor “walking along” while they delivered their care, other students learn and are more comfortable when the preceptor and student codeliver the care. A preceptor stated:

…*Just gradually. I guide them through slowly so that they do not feel overwhelmed at first because there's so much at the beginning. But if they just take on little by little they seem to be fine. You know, they just watch at the beginning and then they start doing after have them do little bits at a time. And it's much better for them. By the end they can usually do it all without any, you know, without me having to there every minute. *

Finally, the preceptors were interested in knowing how to communicate a student concern with the respective faculty. If their concern involved an issue of inappropriate behavior or personal appearance in the clinical setting, the preceptors felt comfortable discussing with the student sans the faculty. However, if the concern involved patient safety, they were more likely to involve the faculty, as well as the nursing unit manager, in the resolution process. An experienced preceptor shared:

I *would talk to that student first and hopefully you can resolve whatever problem it is. If it's practice problem or a personal problem and hopefully it can be resolved at that point. If not, then I'll have to contact the clinical instructor and either solve it between the three of us or talk to clinical services how he or she want to handle that. But I would talk to that student first and see if we can try not to involve, you know, as much as possible, but obviously there are some cases that you have to involve the clinical Instructor. *

**Theme 3 (knowing)**

This theme presents information the preceptors shared about knowing when the student was ready to assume increasingly complex patient care assignments. One of the barometers the individual preceptors used to determine this was demonstrated when the student could comprehend the patient's condition and diagnosis beyond the findings of a cursory physical assessment. The preceptors also cued in on the student's nonverbal confidence represented when he or she was able to anticipate and respond to the patients' needs independently. Additionally when verbally quizzed by the preceptor about this or that patient condition, the student was able to respond appropriately to the question thoughtfully and insightfully. A preceptor described a student's behavior by sharing:

*When they go into a patient's room they're not looking around all the time looking to see who's watching over their shoulders, um, watching their hand movements, talking to the people there, and talking to the patients. And how easy they feel like when something is happening that they can just go ahead and start doing something without asking permission. If they see an order from a doctor they just jump right in and go ahead and start doing that instead of waiting to get permission, kind of looking to make sure it's okay. Just seeing them be more independent.*

Knowing how best to have closure with the student was experienced by all preceptors. As the capstone course was ending and closure was imminent, preceptors acknowledged they experienced a sense of impending loss. The majority planned and attended both formal and informal celebrations. Built into the course was time in which both the preceptor and student could reflect on their shared experience and offer constructive feedback to each other.

### 7.3. Findings

Cooperatively, the participants in this study identified the information they believed they needed to be effective when working with senior nursing students during their capstone course. The preceptors spoke of needing to know basic information about the student's past clinical experience and the curriculum of the school of nursing where they were enrolled. The preceptors recognized certain aspects of their role in which they wanted to know how best to meet the learning needs of the student under their tutelage. They also voiced wanting to know how to troubleshoot or problem solve a student issue of concern should one arise. Finally, the preceptors articulated knowing when it was appropriate to give the student greater autonomy when caring for patients and engaging in more complex clinical situations. Also, the preceptors spoke of knowing when the course was concluding and planning for an appropriate closure with the student. The preceptors all shared statements about closure similar to the one below shared by an individual:

…*for the senior students who are getting ready to be done and getting ready to get out on their own and move on in their own careers, I think it's an exciting time. And really by this time ideally you've got a pretty good sense that they are ready to move into that role. If I felt they were not, obviously I would sit back and assess what it is that I need to do or possibly could do to get them ready in their last few weeks to maybe get them over that hump and have them ready*…*a lot of times we'll do a little celebration …it just has a lighter feel and there's excitement building and the last week we usually have some kind of a luncheon or something for them. *

## 8. Strength and Limitations

It is important that the researchers acknowledge the strengths and present the limitations rooted in this study. The strength of the study was the illumination of the learning needs of RNs serving as preceptors for senior nursing students in their capstone course. While much is known about the clinical experiences of students in their capstone course, little information is available that addresses the learning needs of the RNs serving as preceptors. Unless these educational needs are met, the full breadth of the learning experience will not be realized by the students in their capstone course. 

Limitations include the use of preceptors from only two clinical sites, one type of program, and one type of course. This may have had not captured additional ideas if preceptors with experiences from a greater number of sites, nursing programs, and clinical courses were considered. A second limitation was that preceptors were asked to recall from memory what their experience of serving as a preceptor was like for them. While a personal self-report of an experience is an identified strength of qualitative research, it can also be viewed as a limitation. Further research in this area that increases sample size, types of work sites, additional types of courses, and additional geographical representation is warranted. Additionally, research using a mixed-methods approach may provide additional information that would be valuable for the preceptors.

## 9. Conclusion

The purpose of the study was to identify and describe the experience of registered nurses serving in the role of preceptor for senior nursing students during their capstone course. Twelve registered nurses who met the study criteria for inclusion were asked to participate in the study. Data collection was achieved using audiotaped personal interviews with members of the research team. Textual data analysis was conducted using the constant comparative method put forth by Guba and Lincoln [21]. The information gleaned from this study has the potential to inform the scholarly nursing literature about the educational needs of registered nurses in the role of preceptor for a capstone course.

## Supplementary Material

The structured interview guide contained 10 open ended questions. Those questions asked the preceptor to identify what information they needed from the facult and what backgroud preparation was needed. The questions then focused on how the preceptor introducted the student to pateint care expectations, what they looked for indicating the student was read for increasing complex assignments, showing the student the role of non-nursing care providers, and introducing expectations that are not patient care related. The final set of questions asked about confilct resolution strategies, handling closure, interactions with faculty and finished with reflections on their overall expectations as preceptors.

## Figures and Tables

**Table 1:** Participant demographic data.

Range of age in years	28–66 yrs of age	Mean 42.3 years
Gender	9 Female, 3 Male	
Educational Preparation	BSN or BA degree	58% (*n* = 7)
	ADN degree	33% (*n* = 4)
	MSN degree	9% (*n* = 1)
Range of years of experience working as a registered nurse	4–40 yrs	Mean 13.4 years
Range of years working in clinical specialty area	4–29 yrs	Mean 11.2 years
Range of years in current position	2 months–29 years	Mean 7.4 years
Participants who have completed a preceptor educational course		92% (*n* = 11)
Preceptors who hold National Specialty Clinical Certification		58% (*n* = 7)

**Table 2:** Qualitative thematic data.

Theme 1	Needing to know
Theme 2	Wanting to know
Theme 3	Knowing
