# Supplementary material for: Gradually Guiding Nursing Students through Their Capstone Course: Registered Nurse Preceptors Share Their Experiences
Source: Nurs Res Pract. 2011 Apr 14;2011:645125. doi: 10.1155/2011/645125 (PMC3169838; doi:10.1155/2011/645125)
Supplement: Supplementary file 1 — The structured interview guide contained 10 open ended questions. Those questions asked the preceptor to identify what information they needed from the facult and what backgroud preparation was needed. The questions then focused on how the preceptor introducted the student to pateint care expectations, what they looked for indicating the student was read for increasing complex assignments, showing the student the role of non-nursing care providers, and introducing expectations that are not patient care related. The final set of questions asked about confilct resolution strategies, handling closure, interactions with faculty and finished with reflections on their overall expectations as preceptors. [file 645125.f1.pdf]

Appendix A: Demographic Information

Name:____

___

Gender:___

Highest level of Education:_________ Date of graduation from nursing school:_____________

Number of years as an RN: _____ Current Job Title:________________________________

Nursing unit where you work:____

Name of hospital where you work:____

Length of time in current position:_________ Length of time in clinical specialty:_________

List all clinical

certifications:____

Have you participated in clinical nursing research studies before? Yes _______ No __________

How many times have you been a preceptor in the KU Professional Practicum program?_______

Have you served as a preceptor for other capstone nursing students? Yes ________ No_______

How many times have you been a preceptor for a new nursing employee?___________________

What training did you receive prior to becoming a preceptor (please list type of class, number of hours, and approximate dates or none)?____
